# Adiposity and breast, endometrial, and colorectal cancer risk in postmenopausal women: Quantification of the mediating effects of leptin, C‐reactive protein, fasting insulin, and estradiol

**DOI:** 10.1002/cam4.4434

**Published:** 2022-01-20

**Authors:** S. Ghazaleh Dashti, Julie A. Simpson, Vivian Viallon, Amalia Karahalios, Margarita Moreno‐Betancur, Theodore Brasky, Kathy Pan, Thomas E. Rohan, Aladdin H. Shadyab, Cynthia A. Thomson, Robert A. Wild, Sylvia Wassertheil‐Smoller, Gloria Y. F. Ho, Howard D. Strickler, Dallas R. English, Marc J. Gunter

**Affiliations:** ^1^ Clinical Epidemiology and Biostatistics Unit Murdoch Children’s Research Institute Melbourne Australia; ^2^ Centre for Epidemiology and Biostatistics Melbourne School of Population and Global Health The University of Melbourne Melbourne Australia; ^3^ Nutrition and Metabolism Branch International Agency for Research on Cancer (IARC) Lyon France; ^4^ Clinical Epidemiology and Biostatistics Unit Department of Paediatrics University of Melbourne Melbourne Australia; ^5^ The Ohio State University College of Medicine Columbus Ohio USA; ^6^ Hematology/Oncology Kaiser Permanente Downey Downey California USA; ^7^ Department of Epidemiology and Population Health Albert Einstein College of Medicine Bronx New York USA; ^8^ Herbert Wertheim School of Public Health and Human Longevity Science University of California San Diego USA; ^9^ Health Promotion Sciences Mel & Enid Zickerman College of Public Health University of Arizona Cancer Center Tucson Arizona USA; ^10^ Obstetrics and Gynecology, Biostatistics and Epidemiology Oklahoma University Health Sciences Centre Oklahoma City Oklahoma USA

**Keywords:** breast cancer, causal mediation analysis, colorectal cancer, endometrial cancer, estrogens, inflammation, insulin, Obesity

## Abstract

**Background:**

Mechanisms underlying the adiposity–cancer relationship are incompletely understood. We quantified the mediating roles of C‐reactive protein (CRP), leptin, fasting insulin, and estradiol in the effect of adiposity on estrogen receptor (ER)‐positive breast, endometrial, and colorectal cancer risk in postmenopausal women.

**Methods:**

We used a case–cohort study within the Women's Health Initiative Observational Study, analyzed as a cumulative sampling case–control study. The study included 188 breast cancer cases, 98 endometrial cancer cases, 193 colorectal cancer cases, and 285 controls. Interventional indirect and direct effects on the risk ratio (RR) scale were estimated using causal mediation analysis.

**Results:**

For breast cancer, the total effect RR for BMI ≥30 versus ≥18.5–<25 kg/m^2^ was 1.87 (95%CI,1.11–3.13). The indirect effect RRs were 1.38 (0.79–2.33) through leptin and CRP, 1.58 (1.17–2.43) through insulin, and 1.11 (0.98–1.30) through estradiol. The direct effect RR was 0.82 (0.39–1.68). For endometrial cancer, the total effect RR was 2.12 (1.12–4.00). The indirect effect RRs were 1.72 (0.85–3.98) through leptin and CRP, 1.42 (0.96–2.26) through insulin, and 1.24 (1.03–1.65) through estradiol. The direct effect RR was 0.70 (0.23–2.04). For colorectal cancer, the total effect RR was 1.70 (1.03–2.79). The indirect effect RRs were 1.04 (0.61–1.72) through leptin and CRP, 1.36 (1.00–1.88) through insulin, and 1.02 (0.88–1.17) through estradiol. The direct effect RR was 1.16 (0.58–2.43).

**Conclusion:**

Leptin, CRP, fasting insulin, and estradiol appear to mediate the effect of high BMI on cancer risk to different extents, with likely varying degrees of importance between cancers. These insights might be important in developing interventions to modify obesity‐associated cancer risk in postmenopausal women.

**Lay summary**

Mechanisms underlying the effect of adiposity on increased cancer risk are incompletely understood. Here, we quantified the roles of leptin and C‐reactive protein, fasting insulin, and estradiol in explaining the effect of high BMI on estrogen receptor‐positive breast, endometrial, and colorectal cancers in postmenopausal women. We used a novel causal mediation analysis approach and data from a case–cohort study within the Women's Health Initiative Observational Study. The assessed biomarkers explained the increased risk of the three cancers to different extents, and their importance varied between cancers. These insights might be important in developing cancer prevention interventions for postmenopausal women with obesity.

## 1 INTRODUCTION

Excess body fat is a risk factor for several cancers, including estrogen receptor (ER)‐positive postmenopausal breast, endometrial, and colorectal cancers.^1^, ^2^ The mechanisms by which adiposity causes cancer are uncertain, but chronic low‐grade inflammation, hyperinsulinemia, and disturbed metabolism of sex steroid hormones are three hypothesized pathways.^1^

Chronic low‐grade inflammation is a known consequence of excess adiposity. In the obese state, the production of adipocytokines and cytokines is disturbed, characterized by increased secretion of leptin, one of the most abundant adipocytokines with pro‐inflammatory properties.^1^, ^3^ Other less abundant pro‐inflammatory adipocytokines, such as plasminogen activator inhibitor‐1 (PAI‐1), hepatocyte growth factor (HGF), and resistin,^1^ as well as pro‐inflammatory cytokines, including interleukin‐1β (IL‐1β), IL‐6, and tumor necrosis factor‐α (TNF‐α)^1^, ^4^ are also increased, while the production of adiponectin, an counter‐regulatory adipocytokine with anti‐inflammatory properties, is decreased.^1^ This inflammatory status can promote carcinogenesis by enabling cell proliferation, apoptosis, and angiogenesis,^5^ impaired insulin signaling (thereby inducing hyperinsulinemia),^6^ disturbed bioavailable sex steroid hormone levels through enhanced aromatase expression,^7^ and downregulating of sex hormone‐binding globulin (SHBG) production.^8^ Individuals with obesity are also more likely to be insulin resistant and have chronic hyperinsulinemia.^9^ Insulin has mitogenic and anti‐apoptotic effects.^1^, ^10^, ^11^ Hyperinsulinemia might also affect cancer risk through increasing bioavailable insulin‐like growth factor 1 (IGF‐1) levels. Bioavailable IGF‐1 is growth promoting,^11^, ^12^ and increases bioavailable estrogens through enhanced aromatase expression,^13^ and downregulated SHBG production.^8^ Estrogens have proliferative effects on breast and endometrial tissues,^1^ while there is some evidence that they might have anti‐proliferative effects on colonic tissue through interaction with ERβ expressed in colonic epithelium.^1^

A better understanding of the role these biomarker pathways play in mediating the effect of adiposity on cancer risk and their potentially differing importance for different cancers might help to identify risk‐reducing targets for individuals with obesity. In the present study, we made use of an existing case–cohort study of obesity‐related biomarkers and breast, endometrial, and colorectal cancers among postmenopausal women within the Women's Health Initiative Observational Study (WHI‐OS).^14^, ^15^, ^16^, ^17^ The study is one of the few that has measurements for a range of biomarkers reflecting the three pathways discussed above, including inflammatory biomarkers (including leptin and C‐reactive protein (CRP)), fasting levels of insulin, and estradiol, and provides a unique resource for quantifying and comparing the role of these biomarkers in explaining the effect of adiposity on the three cancers. The most commonly used approach in biomedical research to investigate the mediating roles of biomarkers has been the *difference method*. This method would entail estimating the adiposity–cancer association first without, then with the biomarker (mediator) in the regression model, and taking the difference between the coefficients from the two models as an estimate of the mediating (indirect) effect.^18^ Although straightforward, this approach relies on stringent assumptions and in particular does not extend to situations where multiple mediators affect each other.^18^ As described above, however, it appears unlikely that biomarkers involved in the adiposity–cancer relationship exert their influences independently of each other. Recently, mediation analysis methodology has undergone an overhaul, based on the causal inference literature. The resulting framework, broadly referred to as causal mediation analysis, overcomes some of the limitations of the more commonly applied difference method. More importantly, extensions of this framework allow assessment of mediation in settings with multiple dependent mediators.^19^ Here, we performed causal mediation analysis using a simulation‐based, regression‐standardization mediation analysis approach^20^, ^21^, ^22^ that permitted us to take the dependencies between biomarkers into account and to quantify the so‐called “interventional indirect (mediating) effects” of CRP/leptin, fasting insulin, and estradiol in explaining the effect of adiposity on ER‐positive breast, endometrial, and colorectal cancers in postmenopausal women.

## 2 METHODS

The Women's Health Initiative Observational Study (WHI‐OS) included 93,676 postmenopausal women aged 50–79 years old, recruited from 40 centers across the United States from 1993 to 1998.^23^ At baseline, participants provided written informed consent and completed a questionnaire on demographic and lifestyle factors, medical history, and history of medication use. Height, weight, and waist and hip circumferences were measured during a physical examination, when fasting blood samples were also collected, processed, and plasma and serum stored at −80°C.^23^ Cancer cases were ascertained by annual self‐administered questionnaires and confirmed via centralized review of pathology reports, discharge and consultant summaries, operative and radiology reports, and tumor registry abstracts.^24^ For the present study, follow‐up ended on 29 February 2004. By then 1.6% of all participants were lost to follow‐up and 4.7% had died.

### 2.1 Participants

We made use of an existing case–cohort study derived from the baseline WHI participants, which investigated inflammation, fasting insulin, and estradiol and the risk of endometrial, breast, and colorectal cancers.^14^, ^15^, ^16^ All women diagnosed with any of these three cancers by 29 February 2004 and a random sample of all women in the cohort, regardless of their subsequent cancer diagnosis status (sub‐cohort), were included in the case–cohort study. Women were not included if they had less than 1 year of follow‐up, were diagnosed with endometrial, breast, or colorectal cancer in the first year after enrollment, or were taking diabetes medication at baseline.^14^, ^15^, ^16^

For the present analysis, we treated these data as if the design followed an unmatched case–control study with cumulative sampling because methods for mediation analysis for estimation of interventional indirect effects have not been adapted to the case–cohort design. However, since outcome was rare in the sub‐cohort and a small proportion of participants died or were lost to follow‐up, we expected that analyzing the study as cumulative sampling case–control study to not materially influence the results.^25^

Our analytic dataset included women diagnosed with ER‐positive breast, endometrial, or colorectal cancer in the original case–cohort study as cases, and women in the sub‐cohort who had not been diagnosed with those cancers, and had not died or were not lost to follow‐up by the end of follow‐up period as controls, if they met the following additional eligibility criteria (Table 1).

**TABLE 1:** Number of women affected and unaffected with ER‐positive breast cancer, endometrial cancer, and colorectal cancer in the original case–cohort study, number excluded for the present analysis, and the final number in the analytic dataset for this study

Women included in the original case–cohort study	Breast cancer cases *N* = 875	Endometrial cancer cases *N* = 284	Colorectal cancer cases *N* = 456	Sub‐cohort members *N* = 839
	ER‐positive breast cancer cases *N* = 554			
Constructing the control group from the sub‐cohort for the cumulative sampling case–cohort analysis				
Exclude women in the sub‐cohort diagnosed with endometrial, breast, or colorectal cancer by the end of follow‐up*				28
Exclude women in the sub‐cohort who died or were lost to follow‐up by the end of follow‐up				54
Women considered for the cumulative sampling case–cohort analysis	ER‐positive breast cancer cases *N* = 554	Endometrial cancer cases *N* = 284	Colorectal cancer cases *N* = 456	Controls *N* = 757
Imposed exclusion criteria				
Used hormone therapy at baseline/ unknown hormone therapy use	303	133	161	347
Diagnosed with diabetes at baseline/unknown diabetes status	4	4	5	4
History of any cancer diagnosis at baseline (except keratinocyte skin cancer)	23	34	65	71
With BMI<18.5 kg/m^2^	1	1	2	3
Missing baseline BMI	3	1	5	15
Missing any biomarker data	17	11	18	17
Missing confounder data	15	2	7	15
Women included in the cumulative sampling case–cohort analysis	*N* = 188	*N* = 98	*N* = 193	*N* = 285 *N* = 198 without history of hysterectomy at baseline eligible as controls for endometrial cancer analysis

Abbreviations: BMI, body mass index; ER, estrogen receptor; kg/m^2^, kilograms per meter squared

* These women were included in the analysis as cases.

We additionally excluded cases and controls if at baseline they were current hormone therapy users or reported unknown hormone therapy use, reported a history of diagnosis of diabetes or unknown diabetes status, had a history of any cancer diagnosis (except keratinocyte skin cancer), or had BMI <18.5 kg/m^2^. Women with diabetes or unknown diabetes status were excluded because both diabetes and diabetes medication use might change the adiposity–insulin signaling relationship.^26^ Women were excluded if there were missing values for baseline BMI, confounders (see *Confounder Selection*), or biomarkers (see *Biomarkers*). Only for endometrial cancer, controls with history of hysterectomy at baseline were also excluded (Table 1).

The analytic dataset included 188 ER‐positive breast cancer cases (median follow‐up 3.6 years [interquartile range 2.4–4.8]), 98 endometrial cancer cases (median follow‐up 3.3 years [interquartile range 2.6–4.8]), 193 colorectal cancer cases (median follow‐up 3.8 years [interquartile range 2.7–5.3]), and 285 controls (198 without history of hysterectomy at baseline and eligible as controls for endometrial cancer) (median follow‐up 7.0 years [interquartile range 5.9–7.9]) (Table 1).

### 2.2 Biomarkers

Biomarker measurements were performed on samples collected at baseline. A range of biomarkers of inflammation, including adiponectin, resistin, HGF, PAI‐1, TNF‐α, IL‐6, leptin, and CRP were measured in plasma samples of participants selected for the original case–cohort study.^14^, ^15^, ^16^ For the present study, we only included leptin, an adipocytokine with pro‐inflammatory properties,^1^, ^3^ and CRP, a general marker of inflammation, as measures of inflammation because prior analyses using these data suggested that, within WHI‐OS, the evidence was strongest for associations between CRP and breast^14^ and endometrial cancers^16^ and leptin and colorectal cancer.^15^ Leptin was measured with MILLIPLEX Human Adipokine Panel‐B (interassay coefficients of variation (CV) 9%) and CRP with latex‐enhanced immunonephelometry on the Behring Nephelometer II analyzer (Behring Diagnostics, San Jose, CA; CV 4%).^14^, ^15^, ^16^ Fasting insulin and estradiol were measured in serum in duplicates. Tests with, respectively, >20% and >10% CV were repeated.^27^, ^28^, ^29^ Estradiol was measured using the Vitros‐Eci Immunodiagnostic Assay (Ortho‐Clinical Diagnostics).

### 2.3 Statistical analysis

Body mass index (BMI) was the measure of adiposity (exposure) and to assist with interpretation of the interventional indirect effects (see *Causal mediation analysis)* it was included as a categorical variable in all analyses. Women were categorized based on the WHO cutoff values (BMI≥18.5–<25 kg/m^2^, ≥25–<30, and ≥30 kg/m^2^).^30^ The assessed mediators (biomarkers) were leptin and CRP, fasting insulin, and estradiol, which were log‐transformed and included as continuous variables in all analyses. Separate analyses for each outcome (diagnosis of ER‐positive breast, endometrial, and colorectal cancers) were performed.

We did the following analyses: (i) preliminary analyses to investigate BMI–biomarker associations within the controls (i.e, exposure–mediator association); (ii) preliminary analyses to investigate the BMI–cancer and biomarker–cancer associations (i.e, exposure–outcome and mediator–outcome associations); and iii) causal mediation analyses to decompose the total BMI–cancer association into the interventional indirect (mediated) and direct effects.^20^, ^21^, ^22^, ^31^ All analyses were complete case and performed in Stata version 15.^32^

#### 2.3.1 Confounder selection and assumptions

**FIGURE 1:**
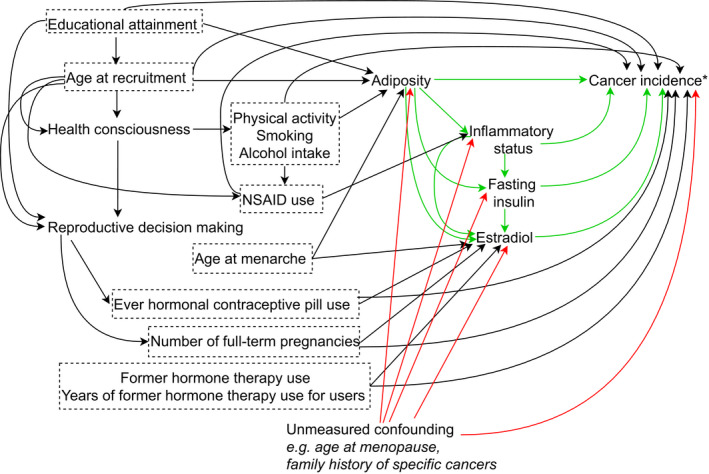
Assumed causal diagram for quantifying the mediating effects of inflammatory status, fasting insulin, and estradiol on adiposity–cancer (estrogen receptor‐positive breast cancer, endometrial cancer, and colorectal cancer) association in postmenopausal women. Abbreviations: NSAID nonsteroidal anti‐inflammatory drug. *To avoid redundancy, a single causal diagram has been presented for the three cancers included in this study (i.e., estrogen receptor‐positive breast cancer, endometrial cancer, and colorectal cancer). Also, to simplify the diagram, we only included the arrows that were sufficient to flag a variable as a common cause (i.e., confounder) of exposure–outcome, exposure–mediator, or mediator–outcome. Therefore, this is not a causal diagram per se. It is assumed that all covariates were pre‐exposure confounders. The diagram was developed with reference to the literature and expert opinion. The green arrows depict the direct and indirect pathways we were interested in, the red arrows the pathways that might have introduced bias due to unmeasured confounding, and the black arrows the backdoor pathways that were blocked by conditioning on the measured confounders (conditioning signaled by dashed boxes around these variables which were adjusted for in all analyses)

The causal diagram in Figure 1 was used as the conceptual model guiding all the analyses and adjustments across the analyses. Analysis investigating BMI–biomarker associations (analysis i) relied on the assumption that there was no residual confounding of the exposure–mediator associations given the pre‐exposure confounders adjusted for. Analysis investigating BMI–cancer association (analysis ii) relied on the assumption that the there was no residual confounding of the exposure–outcome associations given the pre‐exposure confounders adjusted for. And analyses investigating biomarker–cancer association (analysis ii) relied on the assumption that the there was no residual confounding of the biomarker/mediator–outcome associations given the pre‐exposure confounders, exposure, and antecedent biomarkers.^18^ Pre‐exposure confounders were identified a priori based on existing evidence^33^, ^34^, ^35^, ^36^ and included baseline age, educational attainment (some college or less, college and above), alcohol intake (never or former, <1 drink per week, and ≥1 drink per week), ever smoker, physical activity (total metabolic equivalents per week), ever nonsteroidal anti‐inflammatory drug use for ≥2 weeks, age at menarche, parity, ever use of oral contraceptives, former use of hormone therapy and duration of hormone therapy use for former users, age at menopause, and for each cancer, and family history of the specific cancer (Figure 1). Information on all these potential pre‐exposure confounders was collected at baseline. Due to large number with missing data, final analyses did not include age at menopause (9% missing), family history of breast cancer (53% missing), family history of endometrial cancer (55% missing), and family history of colorectal cancer (8% missing) on the basis that all were weakly related with adiposity measures and biomarkers (Table S1).

##### Preliminary analyses of BMI–biomarker associations

For the controls, for each biomarker, the geometric mean ratio (GMR) and 95% confidence intervals (CIs) were estimated in relation to BMI using a linear regression model fitted to the log‐transformed biomarker variable and adjusted for the pre‐exposure confounders.

##### Preliminary analyses of BMI–cancer and biomarker–cancer associations

Risk ratios (RRs) and 95% CIs were estimated from the logistic regression models for the BMI–cancer and biomarker–cancer associations from the logistic regression models based on the formula $risk=\frac{1}{\left(1+\exp\left(‐\log\left(\mathrm{odds}\right)\right)\right)}$.^37^ Prior to calculating risks, the term $\log(\frac{1}{sampling\;fraction\;of\;controls})$ was subtracted from the constant term to take the cumulative case–control design into account.^38^ All models were conditional on the pre‐exposure confounders. Models for biomarkers also included BMI, as it might have confounded the biomarker–cancer association. Additionally, models for fasting insulin also included CRP (as a proxy for inflammatory status) to take its potential confounding effect on fasting insulin–cancer association into account (Figure 1). Similarly, models for estradiol additionally included CRP and fasting insulin as potential confounders (Figure 1). For leptin and CRP, we also present results from models additionally adjusted for fasting insulin, because there is weak evidence suggesting that insulin resistance in the obese state might be sufficient to induce inflammation.^39^ (In Figure 1 the arrow is from inflammatory status to insulin, because although evidence is mixed, this direction is in line with the more convincing evidence).

The linearity of the mediator–cancer associations was investigated by fitting the biomarker variables as restricted cubic splines (with two degrees of freedom).

##### Causal mediation analysis

The mediation analysis decomposes the total effect of BMI on cancer risk into indirect effects through the biomarkers, and a direct effect, which is the part of the effect that remains unexplained by the biomarkers. We followed the decomposition approach proposed by Moreno‐Betancur et al. ^21^ to estimate the RRs for indirect effect through (1) leptin and CRP, interpreted as the average change in cancer risk in women under obesity if we intervened on leptin and CRP levels to change their joint distribution from what it is under obesity, to what it is under normal weight, while the joint distribution of fasting insulin and estradiol remained at its distribution under obesity, (2) fasting insulin, interpreted similarly as the average change in cancer risk in women with obesity if we intervened on fasting insulin to change its distribution to what it is under normal weight, while the joint distribution of leptin, CRP, and estradiol remained as under obesity, and (3) estradiol, interpreted similarly as the average change in cancer risk in women with obesity if we intervened on estradiol to change its distribution to what it is under normal weight, and (4) direct effect not through the assessed biomarkers, interpreted as the average change in cancer risk comparing women with obesity versus normal weight, while the joint distribution of leptin, CRP, fasting insulin, and estradiol remained as under normal weight.^21^ All the effects were averaged over the distribution of the confounders in the control group.

A simulation‐based, regression‐standardization approach was used to estimate the indirect and direct effects, where the expected outcomes under different scenarios were averaged over mediator distributions based on a large number of Monte Carlo draws (~1000 times the sample size^22^), with the distributions of the confounders set to their observed distributions in the sample.^22^ Standard errors (and 95%CIs) were estimated using 1000 bootstrap samples. The approach used builds on a series of regression models listed in Table S1. ^21^, ^22^

## 3 RESULTS

At baseline, compared with the control group, women with ER‐positive breast cancer had fewer term pregnancies and a smaller proportion of them were never or former drinkers. Baseline characteristics were largely comparable for endometrial cancer cases and controls. For colorectal cancer, a smaller proportion of cases had ever used oral contraceptives or were former hormone therapy users at baseline compared with controls (Table 2). For all cancer sites, cases were more likely to have BMI≥30 kg/m^2^, and higher median values for leptin, CRP, and fasting insulin relative to controls. For breast and endometrial cancers, cases also had higher median values for estradiol compared with controls (Table 2).

**TABLE 2:** Baseline characteristics of women included in the analytic dataset

N (%) or median (IQR)	ER‐positive breast cancer cases	Endometrial cancer cases	Colorectal cancer cases	Controls	Controls without history of hysterectomy at baseline
	*N* = 188	*N* = 98	*N* = 193	*N* = 285	*N* = 198
Age at baseline‐years	66.0 (60.0–70.0)	64.5 (60.0–71.0)	67.0 (61.0–72.0)	64.0 (58.0–69.0)	63.0 (57.0–69.0)
Educational attainment					
Some college or less	99 (53)	51 (52)	120 (62)	163 (57)	109 (55)
College and above	89 (47)	47 (48)	73 (38)	122 (43)	89 (45)
Alcohol intake					
Never or former drinker	39 (21)	30 (31)	54 (28)	93 (33)	61 (31)
<1 drink per week	53 (28)	33 (34)	70 (36)	93 (33)	69 (35)
≥1 drink per week	96 (51)	35 (36)	69 (36)	99 (35)	68 (34)
Ever smoked	91 (48)	52 (53)	95 (49)	134 (47)	100 (51)
Physical activity‐total METs per week	7.5 (2.0–17.5)	11.0 (3.4–18.8)	8.5 (2.5–16.3)	9.3 (2.5–19.8)	9.8 (3.5–21.0)
Used NSAIDs 2 weeks or more	64 (34)	30 (31)	56 (29)	90 (32)	62 (31)
Age at menarche‐years	13.0 (12.0–13.0)	13.0 (11.0–13.0)	13.0 (12.0–13.0)	13.0 (12.0–13.0)	13.0 (12.0–13.0)
Number of term pregnancies	2.0 (1.0–4.0)	3.0 (1.0–3.0)	3.0 (1.0–4.0)	3.0 (2.0–4.0)	3.0 (2.0–4.0)
Ever used oral contraceptives	58 (31)	37 (38)	50 (26)	104 (36)	77 (39)
Former hormone therapy use	46 (24)	25 (26)	38 (20)	82 (29)	47 (24)
Duration of former hormone therapy use in ever users‐months	2.8 (1.0–6.0)	1.0 (0.5–6.3)	2.0 (1.0–5.0)	3.0 (0.6–9.3)	1.5 (0.6–5.0)
Adiposity measures					
Body mass index‐kg/m^2^					
[18.5–25]	58 (31)	33 (34)	58 (30)	100 (35)	72 (36)
[25–30]	69 (37)	24 (24)	66 (34)	110 (39)	75 (38)
≥30	61 (32)	41 (42)	69 (36)	75 (26)	51 (26)
Body mass index‐kg/m^2^	27.1 (24.1–31.1)	28.2 (24.1–34.8)	27.5 (24.6–31.3)	26.7 (23.8–30.3)	26.6 (23.5–30.2)
Biomarkers					
Leptin‐ng/Ml	16930.1 (9265.9–29655.4)	20275.4 (8429.7–38645.9)	16923.2 (8635.1–30281.6)	14935.2 (7732.1–26344.5)	14833.9 (7597.4–24636.2)
C‐reactive protein‐µg/mL	1.9 (0.9–3.6)	2.2 (1.1–4.7)	2.0 (0.8–4.0)	1.4 (0.6–3.3)	1.4 (0.6–3.3)
Insulin‐µIU/mL	6.5 (4.5–10.4)	6.5 (4.4–10.3)	7.1 (4.2–11.0)	5.4 (3.7–8.6)	5.2 (3.6–8.1)
Estradiol‐pg/mL	12.5 (8.4–17.0)	13.4 (9.1–21.0)	11.0 (7.6–16.0)	11.0 (6.7–16.0)	10.5 (6.5–15.0)

Abbreviations: ER, estrogen receptor; IQR, interquartile range; MET, metabolic equivalents; ng/mL, nanograms per milliliter; pg/mL, picograms per milliliter; uIU/mL, micro‐IU per milliliter; μg/mL, micrograms per milliliter

### 3.1 Preliminary analyses of BMI–biomarker associations

Positive association was observed for leptin, CRP, fasting insulin, and estradiol, when comparing women with obesity or overweight versus normal weight (Table 3).

**TABLE 3:** Adiposity–biomarker association; analysis limited to controls; *n* = 285

	Geometric mean ratio (95% confidence interval)	
	Body mass index, kg/m^2^	
Biomarker	(25–30) vs. (18.5–25)	≥30 vs. (18.5–25)
Leptin‐ng/mL	2.72	4.50
	(2.25 to 3.28)	(3.62 to 5.59)
CRP‐µg/mL	1.82	3.37
	(1.34 to 2.48)	(2.37 to 4.80)
Fasting Insulin‐µIU/mL	1.59	2.15
	(1.37 to 1.84)	(1.81 to 2.55)
Estradiol‐pg/mL	1.12	1.44
	(0.94 to 1.34)	(1.17 to 1.76)

Note

All analyses were complete case. Models were adjusted for age at baseline, ethnicity, educational attainment, alcohol intake, ever smoked, physical activity, NSAID use, age at menarche, parity, oral contraceptive use, former hormone therapy use, and duration of former hormone therapy use.

Taking estradiol as an example, the geometric mean ratio of 1.44 could be interpreted as 44% increase in (or 1.44 times higher) geometric mean of estradiol for postmenopausal women with BMI ≥30 kg/m^2^ compared with those with BMI ≥18.5–<25 kg/m^2^.

### 3.2 Preliminary analyses of BMI–cancer and biomarker–cancer associations

For ER‐positive breast cancer, positive RRs were observed for women with BMI ≥30 versus ≥18.5–<25 kg/m^2^ (adjusted RR 1.87; 95%CI, 1.11–3.13). There were positive associations with fasting insulin and estradiol, with the larger RR observed for fasting insulin (RR for doubling in fasting insulin levels and adjusted for confounders, BMI, and CRP 1.56; 95%CI, 1.18–2.04). There was also evidence for positive associations with leptin and CRP, and the associations were somewhat attenuated after adjusting for insulin (Table 4).

**TABLE 4:** Adiposity–cancer and biomarker–cancer associations (adjusted risk ratios and 95% confidence intervals).

	Breast cancer				Endometrial cancer				Colorectal cancer			
Body mass index, kg/m^2^ (Adj for baseline confounders)												
(25–30) vs. <25	1.07	(0.67 to 1.70)			0.69	(0.36 to 1.30)			0.96	(0.60 to 1.52)		
≥30 vs. <25	1.87	(1.11 to 3.13)			2.12	(1.12 to 4.00)			1.70	(1.03 to 2.79)		
Biomarker, Per doubling concentration												
				*P* value‐evidence against linearity*				*P* value‐evidence against linearity*				*P* value‐evidence against linearity*
Leptin												
Adj for baseline confounders and BMI	1.24	(1.02 to 1.50)		0.75	1.38	(1.06 to 1.81)		0.14	1.09	(0.90 to 1.33)		0.81
Add adj for fasting insulin	1.13	(0.92 to 1.38)			1.27	(0.95 to 1.68)			1.01	(0.82 to 1.25)		
CRP												
Adj for baseline confounders and BMI	1.15	(1.01 to 1.32)		0.06	1.22	(1.03 to 1.44)		0.39	1.05	(0.93 to 1.20)		0.82
Add adj for fasting insulin	1.11	(0.97 to 1.28)			1.18	(0.99 to 1.40)			1.03	(0.90 to 1.17)		
Fasting insulin												
Adj for baseline confounders and BMI	1.61	(1.23 to 2.10)		0.45	1.55	(1.10 to 2.20)		0.34	1.34	(1.04 to 1.73)		0.48
Add adj for CRP	1.56	(1.18 to 2.04)			1.46	(1.03 to 2.08)			1.33	(1.02 to 1.72)		
Estradiol												
Adj for baseline confounders and BMI	1.25	(1.01 to 1.55)		0.08	1.72	(1.27 to 2.32)		0.80	1.04	(0.84 to 1.30)		0.24
Add adj for CRP and fasting insulin	1.22	(0.97 to 1.52)			1.66	(1.22 to 2.26)			1.03	(0.83 to 1.29)		

Note

All analyses were complete case. Models were adjusted for age at baseline, educational attainment, alcohol intake, ever smoked, physical activity, NSAID use, age at menarche, parity, oral contraceptive use, former hormone therapy use, and duration of former hormone therapy use. All models for biomarkers were additionally adjusted for body mass index.

Abbreviations: BMI, body mass index; CRP, C‐reactive protein.

* *P* values from likelihood ratio test

For endometrial cancer, a positive association was observed with BMI ≥30 versus ≥18.5–<25 kg/m^2^ (RR 2.12; 95%CI, 1.12–4.00). There were positive associations with leptin and CRP, which were slightly attenuated after adjusting for insulin, and with fasting insulin, and estradiol. The strongest association was observed for estradiol (RR for doubling in estradiol levels and adjusted for confounders, BMI, CRP, and insulin 1.66; 95%CI, 1.22–2.26) (Table 4).

For colorectal cancer, the adjusted RR was 1.70 (95%CI, 1.03–2.79) for BMI ≥30 versus ≥18.5–<25 kg/m^2^. Of the biomarkers, a positive association was only observed for fasting insulin (RR for doubling in fasting insulin levels and adjusted for confounders, BMI, and CRP 1.33; 95%CI, 1.02–1.72) (Table 4).

For all cancer sites, there was no strong evidence for departure from linearity for any of the biomarkers (Table 4). For each cancer site, we performed mediation analysis comparing BMI ≥30 versus ≥18.5–<25 kg/m^2,^ as there was no evidence for association comparing BMI ≥25–<30 kg/m^2^ versus ≥18.5–<25 kg/m^2^.

### 3.3 Mediation analysis

For ER‐positive breast cancer, comparing women with BMI ≥30 versus ≥18.5–<25 kg/m^2^, we estimated a positive indirect effect through leptin and CRP (RR 1.38) but with high uncertainty (95%CI, 0.79–2.33), and a stronger indirect effect through fasting insulin (RR 1.58; 95%CI, 1.17–2.43). The indirect effect through estradiol was weaker (RR 1.11; 95%CI, 0.98–1.30). The RR for direct effect was 0.82 (95%CI, 0.39–1.68) (Table 5).

**TABLE 5:** Indirect (mediated) and direct effects comparing women with obesity versus normal weight

			BMI, kg/m^2^ ≥30 vs. (25–30)
	#	Effects	Risk ratio (95% CI)
ER‐positive breast cancer	1	Interventional indirect effect through leptin and CRP	1.38
			(0.79 to 2.33)
	2	Interventional indirect effect through fasting insulin	1.58
			(1.17 to 2.43)
	3	Interventional indirect effect through estradiol	1.11
			(0.98 to 1.30)
	4	Interventional direct effect	0.82
			(0.39 to 1.68)
Endometrial cancer	1	Interventional indirect effect through leptin and CRP	1.72
			(0.85 to 3.98)
	2	Interventional indirect effect through fasting insulin	1.42
			(0.96 to 2.26)
	3	Interventional indirect effect through estradiol	1.24
			(1.03 to 1.65)
	4	Interventional direct effect	0.70
			(0.23 to 2.04)
Colorectal cancer	1	Interventional indirect effect through leptin and CRP	1.04
			(0.61 to 1.72)
	2	Interventional indirect effect through fasting insulin	1.36
			(1.00 to 1.88)
	3	Interventional indirect effect through estradiol	1.02
			(0.88 to 1.17)
	4	Interventional direct effect	1.16
			(0.58 to 2.43)

Note

Models were adjusted for age at baseline, educational attainment, alcohol intake, ever smoked, physical activity, NSAID use, age at menarche, parity, oral contraceptive use, former hormone therapy use, and duration of former hormone therapy use.

For all cancers, the product of the indirect and direct effects (effects 1 to 4), which can be taken as the estimate of the total effect of having obesity versus normal weight on cancer risk, is close to, but not exactly the same as, the estimated RRs reported in Table 4. The small difference is because the parametric assumptions made by the models used to estimate the effects reported in Table 4 (logistic regression model of the outcome conditional on exposure and confounders) is different from the assumptions made by models used to estimate the effects reported in Table 5 (logistic regression model of the outcome conditional on exposure, all mediators, and confounders).

For endometrial cancer, we estimated a positive indirect effect through estradiol (RR 1.24; 95%CI, 1.03–1.65), and a stronger indirect effect through fasting insulin (RR 1.42; 95%CI, 0.96–2.26). The point estimate was indicative of even a stronger indirect effect through leptin and CRP (RR 1.72), but there was high uncertainty around the estimated effect (95%CI, 0.85–3.98). The RR for direct effect was 0.70 (95%CI, 0.23–2.04) (Table 5).

For colorectal cancer, the point estimate suggested negligible indirect effects through leptin and CRP (RR 1.04; 95%CI, 0.61–1.72) and estradiol (RR 1.02; 95%CI, 0.83–1.17), with high uncertainty around the estimated effects, but there was a positive indirect effect through fasting insulin (RR 1.36; 95%CI, 1.00–1.88) The RR for direct effect was 1.16 (95%CI, 0.58–2.43) (Table 5).

## 4 DISCUSSION

In this study of postmenopausal women, we quantified the mediating effects of leptin and CRP, fasting insulin, and estradiol in explaining the associations between obesity and ER‐positive breast, endometrial, and colorectal cancers. The results indicated that the contribution of the assessed biomarkers to the obesity–cancer associations might differ across cancer sites. For ER‐positive breast cancer, the largest mediating effect was observed through fasting insulin, followed by leptin and CRP, albeit with less certainty, and a smaller indirect effect through estradiol. For endometrial cancer, there was positive mediating effect through estradiol, while with less certainty, the estimates were also indicative of possible mediating effects through insulin, and leptin and CRP. For colorectal cancer, there was evidence for mediating effect through fasting insulin, while the point estimates for indirect effects through leptin and CRP, and estradiol were essentially null. For the three cancers, the 95% CIs around the estimated direct effects were wide and we were unable to make definitive judgments about the direction and magnitude of the effect of obesity on cancer risk not explained by the assessed biomarkers.

To our knowledge, this is the first study to simultaneously quantify the mediating role of biomarkers reflecting inflammatory status, hyperinsulinemia, and estradiol in explaining the association between adiposity and risk of ER‐positive breast cancer, endometrial cancer, and colorectal cancer in postmenopausal women using data from the same cohort. An important limitation was the small number of cases and controls for each cancer site.

The causal mediation analysis approach we used allowed us to account for the potential dependencies between the assessed biomarkers. Also, the interpretations of the estimated interventional indirect and direct effects were agnostic to the causal ordering of the mediators.^20^, ^21^, ^22^ This was a considerable advantage compared with other causal mediation analysis approaches that require such an assumption (e.g., the sequential causal mediation analysis approach described in reference^40^), because the existing uncertainty about the causal ordering between pathways did not affect our conclusions.

The estimated mediating roles for the biomarkers depend on the quality of the measurements and the temporal stability of the biomarkers. In general, the biomarker measurements were of good quality (for leptin and CRP the inter‐assay coefficients of variations (CV) were ≤9%, estradiol and insulin tests were performed in duplicates and tests with, respectively, >20% and >10% CV were repeated^27^, ^28^, ^29^). For all biomarkers, we had measurements for a single time point. Previously published data on the intraclass correlation coefficient (ICC) calculated using data collected approximately 3 years aside for leptin, insulin,^41^ and estradiol^42^ or 6 years aside for CRP^43^ suggest that the single time point measurements would likely have reflected the average levels over time (all ICCs were >58%). The extent to which the estimated indirect effects through the assessed biomarkers reflected the role of the pathways they represented would have also depended on how well the biomarkers captured the biological characteristics of each pathway. For example, for the sex steroid hormone pathway, it is unlikely that estradiol on its own would have reflected all aspects of the pathway. Similarly, fasting insulin might not have been the ideal measure of insulin resistance, although it is a good measure for hyperinsulinemia, which is likely the major cancer link.^1^

We excluded cases diagnosed within the first year after baseline to reduce the possibility of cancer having influenced measures of adiposity or biomarkers. However, we cannot rule out reverse causation for the adiposity–biomarker associations. They were assessed at baseline, and we did not adjust for prior biomarker measurements. Our analyses relied on no unmeasured exposure–mediator, exposure–outcome, and mediator–outcome confounding.^18^ Although this assumption is unverifiable and might have been violated to some extent due to unmeasured or residual confounding, effort was made to take all important confounders identified a priori into account in the analyses. In our study, we measured adiposity and biomarkers in middle and older aged postmenopausal women and assessed their associations with cancer in the future. The generalizability of our results is limited to this time window.

Our results generally agree with a previous study that used data from two case–cohort studies within the WHI‐OS, one of which was the same dataset as that used here, and performed a causal mediation analysis to estimate the natural indirect effect of estradiol and fasting insulin in mediating the association of BMI with postmenopausal breast cancer in women not using hormone therapy.^44^ Both studies suggested that fasting insulin and estradiol explained some of the adiposity–postmenopausal breast cancer association, with fasting insulin having a larger role. In the previous study, of the total BMI–breast cancer association (additional cases per 100,000 per 5 kg/m^2^ increment in BMI 50.0; 95%CI, 23.2–76.6), 24% was mediated through estradiol (IE additional cases per 100,000 11.9, 95%CI, 1.7–22.6), with a larger mediating effect (49%) observed for ER‐positive breast cancers. In women without diabetes, of the total effect (additional cases per 100,000 52.0; 95%CI, 12.1–91.3), 66% was mediated through fasting insulin (IE additional cases per 100,000 34.2; 94%CI, 9.4–59.0).^44^ Contrary to the previous study that assumed independence between estradiol and fasting insulin, our analysis also allowed for potential dependence between the assessed biomarkers. In another causal mediation analysis of the adiposity–ER‐positive postmenopausal breast cancer association (RR for BMI>30 vs. ≤25 kg/m^2^ 1.75; 95%CI, 1.05–2.91), conducted as a case–control study within the Melbourne Collaborative Cohort Study (MCCS), 72% of the effect was explained by free estradiol (RR 1.56; 95% CI, 1.11–2.19) and 28% by fasting insulin (RR 1.12; 95%CI, 0.68–1.84).^45^ The weaker mediating effect observed for estradiol in the present study might be due to lack of information on free estradiol, which, compared with total estradiol, is likely a more important mediator of the adiposity–breast cancer association.^46^ In line with the weaker mediating effect observed for insulin in the MCCS, the reported insulin–postmenopausal breast cancer association was weaker in that study (HR per doubling concentration 1.00; 95%CI, 0.65–1.53), compared with what we observed here and with reported in a recent Mendelian randomization (MR) study (OR per genetically determined one standard deviation in insulin level 2.07; 95%CI, 1.32–3.23).^47^ Different assays used in the MCCS and WHI‐OS, different distributions of confounding factors in the studies, and/or chance (the 95%CIs from MCCS, WHI‐OS, and the MR study did overlap) might all have contributed.

In a study of six European cohorts that used causal mediation analysis to explain the effect of adiposity on endometrial cancer risk in pre‐ and postmenopausal women,^48^ of the total effect of BMI on endometrial cancer risk (hazard ratio (HR) per 5 kg/m^2^ 1.50; 95% CI, 1.43–1.57), the indirect effect through triglyceride glucose product (TyG index, used as a proxy measure for insulin resistance) was essentially null (HR per 5 kg/m^2^ 1.01; 95% CI, 0.99–1.03), suggesting that the biomarker did not explain any of the total BMI–endometrial cancer risk association. The study also observed no evidence of an association between TyG and endometrial cancer risk (HR per 1 standard deviation (SD) increase in TyG 1.04; 95% CI, 0.98–1.11). In our study, we observed an association between fasting insulin and endometrial cancer risk, which was in line with results from a Mendelian randomization study (OR for decrease of one SD in genetically determined fasting insulin level 2.34; 94%CI, 1.06–5.14)^49^ and a suggestion for a mediating effect through fasting insulin, with the point estimate indicating even a larger indirect effect through fasting insulin compared with estradiol. The point estimates from our analysis also implied a mediating effect through leptin and CRP larger than estradiol, but the wide 95%CI around the indirect effect did not allow us to draw conclusions regarding the presence or magnitude of the mediating effect through these biomarkers. In a sequential causal mediation analysis of data from a nested case–control study of postmenopausal women within the European Prospective Investigation into Cancer and Nutrition (EPIC), there was again high uncertainty around the estimated indirect effects. However, based on the point estimates, for the total effect of BMI on endometrial cancer (OR for BMI ≥30 vs. ≥18.5–<25 kg/m^2^ 2.51; 95%CI, 1.26–5.02), the largest indirect effect was through a range of biomarkers of inflammatory status (OR 1.53; 95%CI, 0.89–2.62). A negligible indirect effect was estimated through C‐peptide (used as a proxy measure for insulin resistance) (OR 1.05; 95%CI, 0.88–1.24), and an indirect effect comparable to what we observed for estradiol, through free estradiol and estrone (OR 1.22; 95%CI, 0.89–1.67).^50^ Increased estrogens in obese women are hypothesized to play a key role in explaining the adiposity–endometrial cancer risk association,^51^, ^52^ but based on the EPIC study^50^ and the current analysis, estrogens seem to only partly mediate the effect of adiposity on endometrial cancer risk, suggesting that hyperinsulinemia and inflammation are also likely important.

In the UK Biobank cohort, a sequential causal mediation analysis approach was used to explain the effect of adiposity on colorectal cancer risk in postmenopausal women through CRP, glycated hemoglobin (HbA1c, used as a correlate of insulin resistance), and sex hormone‐binding globulin (SHBG) and testosterone.^53^ Of the total waist circumference–colorectal cancer association (RR for waist circumference >88 vs. ≤80 cm, 1.27; 95%CI, 1.07–1.50), there was evidence for a small mediating effect through CRP (RR 1.08; 95%CI, 0.99–1.17), but not for HbA1c (RR 1.00; 95%CI, 0.98–1.02) or SHBG and testosterone (RR 1.00; 95%CI, 0.92–1.09). In our study, the point estimate indicated an even smaller mediating effect through leptin and CRP, but a stronger mediating effect through fasting insulin, which might be a better marker of disrupted insulin signaling in the obese state than HbA1c.^54^ An interesting finding in the two studies was the essentially null indirect effect observed for the sex steroid hormone pathway, captured by estradiol in the present study, and by SHBG and testosterone in the UK Biobank study.^53^

In summary, among postmenopausal women, we observed that leptin and CRP, fasting insulin, and estradiol might mediate associations of general obesity with ER‐positive breast, endometrial, and colorectal cancers, with suggestion of varying degrees of importance for the biomarkers between the three cancers. Studies with larger sample sizes are needed to further investigate and produce results with more certainty. Ideally, future studies will take advantage of novel causal mediation analysis approaches, similar to the method used in the present study, to appropriately account for dependence between biomarkers and to establish relevant biological targets for future prevention‐based interventions.

## ETHICS STATEMENT

All procedures performed in studies involving human participants were in accordance with the ethical standards of the institutional and/or national research committee and with the 1964 Helsinki declaration and its later amendments or comparable ethical standards. The study was approved by the institutional review boards of all participating institutions. Participants provided written informed consent at enrollment.

## DISCLAIMER

Where authors are identified as personnel of the International Agency for Research on Cancer/World Health Organization, the authors alone are responsible for the views expressed in this article and they do not necessarily represent the decisions, policy, or views of the International Agency for Research on Cancer/World Health Organization.

## CONFLICT OF INTEREST STATEMENT

The authors declare no conflict of interest.

## Supporting information

Table S1

## Data Availability

The data that support the findings of this study are available from the Women's Health Initiative (https://www.whi.org/page/working‐with‐whi‐data).
