# Supplementary material for: Adiposity and breast, endometrial, and colorectal cancer risk in postmenopausal women: Quantification of the mediating effects of leptin, C‐reactive protein, fasting insulin, and estradiol
Source: Cancer Med. 2022 Jan 20;11(4):1145–59. doi: 10.1002/cam4.4434 (PMC8855919; doi:10.1002/cam4.4434)
Supplement: Supplementary file 1 — Table S1 [file CAM4-11-1145-s001.docx]

**Adiposity and breast, endometrial, and colorectal cancer risk in postmenopausal women: quantification of the mediating effects of leptin, C-reactive protein, fasting insulin, and estradiol**

*Running title: The adiposity-cancer link in women*

S Ghazaleh Dashti DDS PhD, Julie A Simpson PhD , Vivian Viallon PhD , Amalia Karahalios PhD , Margarita Moreno-Betancur PhD, Theodore Brasky PhD , Kathy Pan MD, Thomas E Rohan MBBS PhD DHSc, Aladdin H Shadyab PhD , Cynthia A Thomson PhD, Robert A Wild MD PhD, Sylvia Wassertheil-Smoller PhD , Gloria YF Ho PhD, Howard D Strickler MD, Dallas R English PhD , Marc J Gunter PhD

Supplementary material

| Supplementary Table 1 – Regression models used in estimation of interventional indirect and direct effect. The tick marks indicate the specific models that were combined to estimate each effect. | | | | | | |
| --- | --- | --- | --- | --- | --- | --- |
|  | | | Interventional indirect effect through | | | Interventional direct effect |
|  |  |  | Leptin & CRP | Insulin | Estradiol |  |
| Mediator models: | Linear regression of | leptin conditional on BMI (exposure) and confounders | 🗸 | 🗸 | 🗸 | 🗸 |
|  |  | CRP conditional on leptin, BMI and confounders | 🗸 | 🗸 | 🗸 | 🗸 |
|  |  | insulin conditional on BMI and confounders | 🗸 | 🗸 |  |  |
|  |  | estradiol conditional on BMI and confounders |  |  | 🗸 |  |
|  |  | insulin conditional on leptin, CRP, BMI and confounders |  |  | 🗸 | 🗸 |
|  |  | estradiol conditional on insulin, BMI and confounders | 🗸 |  |  |  |
|  |  | estradiol conditional on leptin, CRP, BMI and confounders |  | 🗸 |  |  |
|  |  | estradiol conditional on leptin, CRP, insulin, BMI and confounders |  |  |  | 🗸 |
| Outcome model: | Logistic regression of | outcome conditional on BMI, leptin, CRP, insulin, estradiol, and confounders | 🗸 | 🗸 | 🗸 | 🗸 |
